# TAp73 promotes anabolism

**DOI:** 10.18632/oncotarget.2667

**Published:** 2014-11-13

**Authors:** Ivano Amelio, Alexey A. Antonov, Maria Valeria Catani, Renato Massoud, Francesca Bernassola, Richard A. Knight, Gerry Melino, Alessandro Rufini

**Affiliations:** ^1^ Medical Research Council, Toxicology Unit, Leicester University, Leicester LE1 9HN, UK; ^2^ Biochemistry Laboratory, IDI-IRCCS, University of Rome Tor Vergata, Rome 00133, Italy; ^3^ Molecular Pharmacology Laboratory, Technological University, St-Petersburg, Russia; ^4^ Department of Cancer Studies, Cancer Research UK, Leicester Centre, University of Leicester, Leicester, LE1 7RH, UK

**Keywords:** p73, p53, Metabolism, Warburg effect, cancer

## Abstract

Metabolic adaptation has emerged as a hallmark of cancer and a promising therapeutic target, as rapidly proliferating cancer cells adapt their metabolism increasing nutrient uptake and reorganizing metabolic fluxes to support biosynthesis. The transcription factor p73 belongs to the p53-family and regulates tumorigenesis via its two N-terminal isoforms, with (TAp73) or without (ΔNp73) a transactivation domain. TAp73 acts as tumor suppressor, at least partially through induction of cell cycle arrest and apoptosis and through regulation of genomic stability. Here, we sought to investigate whether TAp73 also affects metabolic profiling of cancer cells. Using high throughput metabolomics, we unveil a thorough and unexpected role for TAp73 in promoting Warburg effect and cellular metabolism. TAp73-expressing cells show increased rate of glycolysis, higher amino acid uptake and increased levels and biosynthesis of acetyl-CoA. Moreover, we report an extensive TAp73-mediated upregulation of several anabolic pathways including polyamine and synthesis of membrane phospholipids. TAp73 expression also increases cellular methyl-donor S-adenosylmethionine (SAM), possibly influencing methylation and epigenetics, and promotes arginine metabolism, suggestive of a role in extracellular matrix (ECM) modeling. In summary, our data indicate that TAp73 regulates multiple metabolic pathways that impinge on numerous cellular functions, but that, overall, converge to sustain cell growth and proliferation.

## INTRODUCTION

p73 is a transcription factor and a member of the p53-family [1–3]. p73 is transcribed in two alternative N-terminal isoforms: the use of an upstream promoter allows expression of transcriptional competent TAp73 isoforms; whereas transcription initiated from a downstream promoter results in the expression of N-terminal truncated isoforms missing the transactivation domain (ΔNp73) [1, 4]. Additional isoforms (α,β,γ,δ) are generated by C-terminal differential splicing [1, 4, 5]. Recently, we demonstrated that TAp73 acts as a tumor suppressor and its depletion results in a tumor prone phenotype [6]. On the contrary, ΔNp73 has oncogenic properties and transformed fibroblasts lacking ΔNp73 fail to grow when injected into immunocompromised mice [7]. The ability of TAp73 to suppress tumor formation depends on several mechanisms, including its ability to induce cell cycle arrest [8] and apoptosis [9–18] through regulation of target genes partially shared with p53 [19–23], such as p21 [24–27], PUMA [28–32] and BAX [33, 34]. These findings notwithstanding, data on humans show that, conversely to p53 [35–41], TAp73 is rarely mutated in cancer and its expression is often retained [1, 3, 42, 43], suggesting a more complex scenario for p73 in cancer.

Over the past decade, the metabolic adaptation of tumor cells has attracted increasing appreciation as main drivers of cancer growth. Cancer cells adopt their metabolism to sustain high rate of cell growth and proliferation and to survive a hostile environment. This alteration is necessary to satisfy metabolic demands including maintenance of elevated cellular ATP levels, active biosynthesis of macromolecules and preservation of the cellular redox equilibrium [44–48]. The change in glucose metabolism known as Warburg effect or aerobic glycolysis is probably the most characterized metabolic adaptation of cancer cells, which show sustained glucose consumption and glycolytic rate. Additional metabolic adaptations have been described for amino acid [49, 50] and lipid metabolism [51, 52], demonstrating an extensive metabolic rewiring that underpins tumorigenesis, and nurturing the idea that therapeutic interventions targeting metabolism could benefit cancer patients [53–58].

Of note, we have recently demonstrated that TAp73 regulates energetic and oxidative metabolism through modulation of mitochondrial function: we showed that depletion of TAp73 impairs activity of the complex IV of the electron transport chain, decreases mitochondrial respiration and intracellular ATP and increases ROS content [59, 60]. Moreover, we unveiled a role for TAp73 in regulating enzymes of serine-glycine metabolism in cancer cells [61] and lipid metabolism in hepatic cells [62]. Shortly after, Du and colleagues showed that glucose-6-phosphate dehydrogenase (G6PD), the rate-limiting enzyme in the pentose phosphate pathway (PPP) is a TAp73 target gene [63]. Though enhanced G6PD expression, TAp73 fuels the PPP leading to increase ribose and NADPH biosynthesis, with the final outcome of reducing cellular ROS and promoting cellular proliferation [63]. These last findings have challenged the common knowledge that TAp73 is a tumor suppressor [1] and suggested that it regulates metabolism in order to sustained cell replication.

Prompted by these results, we attempted to further elucidate the regulation of cellular metabolism by p73 performing high throughput metabolomics study upon ectopic expression of TAp73 in human p53-null osteosarcoma cell lines (SaOs-2). Here we report an interesting role for TAp73 in promoting anabolic metabolism, including increased synthesis of acetyl-CoA, polyamines, and membrane phospholipids. Moreover, our data suggest that TAp73 promotes glycolysis and enhances the Warburg effect. Finally, we uncovered additional regulations impinging on the methyl donor S-adenosyl-methionine (SAM), fatty acids and arginine-proline metabolism.

Overall, our findings demonstrate that TAp73 activates anabolic pathways compatible with proliferation and in line with the report from Du and colleagues. Notwithstanding, we deem the current data insufficient to question the tumor suppressive function of TAp73, which we consistently detect through robust activation of p21 and induction of apoptosis. Our findings should also be interpreted on the light of the multifaceted physiological activities of p73, including regulation of animal aging, senescence, neurodegerative diseases and fertility [59, 64, 65].

## RESULTS

To investigate the effects of TAp73 expression on cellular metabolism, we used human p53/p73 null SaOs-2 osteosarcoma cell line, engineered to overexpress human HA-tagged TAp73 isoforms when cultured in the presence of the tetracycline analog doxycycline (Dox) [29] (Supplemental Figure 1). TAp73 has been reported to halt cell proliferation and to induce apoptosis [1, 16, 28]. Hence, to avoid unwanted effects on metabolic profiling caused by induction of cell cycle arrest or cell death, we carefully investigated the outcome of p73 induction over 72h time course. In our system, expression of both TAp73α C-terminal isoforms reached plateau after 16h of Dox treatment, without any discernible effect on cell cycle distribution (Supplemental Figure 1A and B). Of note, the p73 direct target p21 was also upregulated, indicating that p73 is transcriptionally active at the same time point (Supplemental Figure 1A). Next, we measured induction of cell death as sub-G1 peak. Expression of TAp73α isoform induced considerable apoptosis in late time points, starting at 24h after Dox addition. Importantly, similarly to cell cycle profile, early time points show no difference in cell survival when compared to untreated controls, whereas sustained cell death was observed at later time points (Supplemental Figure 1C).

Taken together, these data indicate that short time induction of TAp73 (8h and 16h) results in robust expression, engagement of transcriptional activity, with no overt effect on cell cycle and cell survival. Consequently, we decided to perform metabolomics profiling in SaOs-2 cell line at early time points (8h and 16h) after induction of TAp73β. We focused on the C-terminal β isoforms because of its stronger transcriptional activity, but affects cell cycle and survival similarly to TAp73α (not shown). The main predictable bias is that a short induction might not be sufficient for completion of elaborated metabolic pathways triggered or inhibited by TAp73. Regardless, we reasoned that changes in a subset of metabolites may suffice in identifying significant metabolic rearrangements triggered by p73.

We used GC-MS and LC-MS-MS platforms for metabolomic studies of TAp73β SaOs-2 Tet-on treated with vehicle or with 2μg/ml doxycycline for 8h and 16h. This approach enabled detection of 292 biochemical compounds, a fraction of which showed significant differences between the different groups (uninduced, 8h and 16h). 15% to 20% of all detected biochemicals displayed statistical significant differences (*p* < 0.05, Welch's, two samples, t-test), between uninduced and induced samples (Table 1), whereas changes in 10% of the compounds approached statistical significance (0.05 < *p* < 0.1).

**Table 1:** Significantly metabolic alterations identified in SaOs-2 cells after TAp73 over expression

SaOs-TAp73β	Dox 8h vs CTRL	Dox 16h vs CTRL	Dox 16h vs Dox 8h
Biochemicals *p* ≤ 0.05	55 (54/1)	42 (34/8)	17 (7/10)
Biochemicals 0.05 ≤ *p* ≤ 0.10	30 (26/4)	28 (15/13)	17 (5/12)

Significantly altered metabolites in SaOs-Tet-on. Red indicates number of upregulated compounds, whereas green indicates downregulation. Following log transformation and imputation with minimum observed values for each compound, Welch's two-sample t-test was used to identify biochemicals that differed significantly between doxycycline-induced sample groups compared to control (no doxycycline), as well as identifying significant biochemical migration as induction progresses from eight to sixteen hours of doxycycline exposure. An estimate of the false discovery rate (*q*-value) is calculated to take into account the multiple comparisons that normally occur in metabolomic-based studies; as *q*-values were reasonable for *p* ≤ 0.05, no *q*-value cutoff was established.

**Figure 1:**
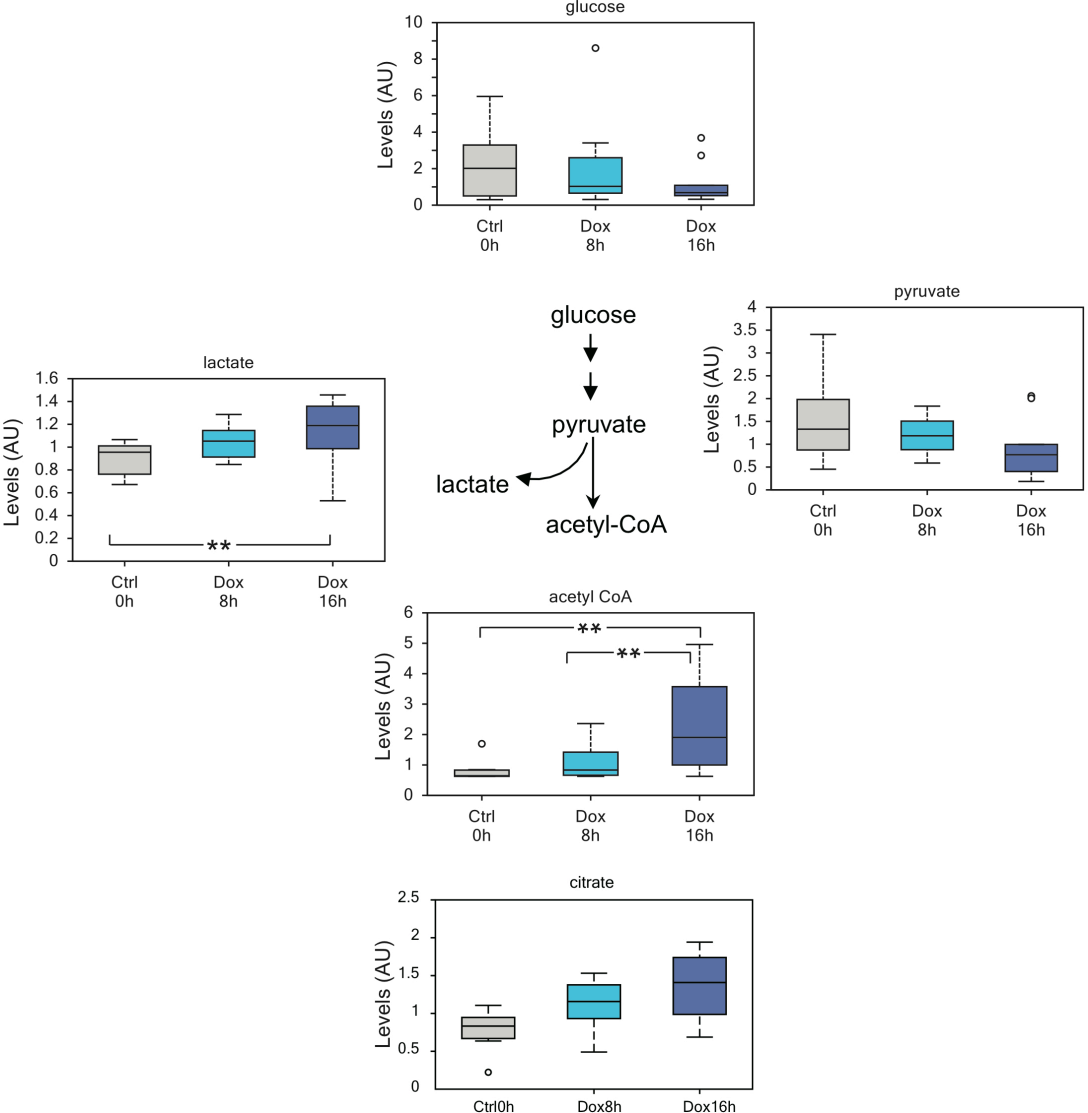
TAp73 enhances glycolytic flux Increased glycolytic flux as suggested by reduced intracellular glucose and concomitant increased lactate and acetyl-CoA. This result suggests that TAp73 expression sustain Warburg effect and might help explaining while its expression is retained in many cancers. The decrease pyruvate levels probably reflect channeling into lactate and acetyl-CoA. Of note, the augmented acetyl-CoA levels may also stem for increased biosynthesis. **p < 0.05; *0.05 < *p* < 0.1.

Glucose metabolism through glycolysis assumes substantial relevance in cancer. One of the hallmarks of many cancer cells is the high rate of glucose consumption and its conversion into lactate even in the presence of oxygen (known as aerobic fermentation, or Warburg effect) [44]. Though still object of intense investigation and debate, the Warburg effect is important to increase energy production and to enrich the cellular milieu with glycolytic intermediates used in biosynthesis pathways necessary to sustain proliferation [44]. Strikingly, expression of p73 produced a decrease in intracellular glucose and pyruvate levels with a concomitant increase in lactate (Figure 1). This suggests that TAp73 is a positive regulator of glycolysis and enhances the Warburg effect. Consistent with enhanced glycolytic flux, we also detected increased amount of acetyl-CoA and citrate (Figure 1). Of note, citrate was the only TCA cycle metabolites to be consistently increased (data not shown). It is possible that 16h are not enough for the build-up of other TCA cycle intermediates, or anaplerotic reactions may contribute to stabilize the TCA cycle activity. Alternatively, citrate could be shunt to fatty acid biosynthesis instead of entering Krebs's cycle, although we did not observed increased free fatty acids at 16h post-induction. On the other hand, the increase in acetyl-CoA, correlated with an overall increase in the biosynthesis of coenzyme A (CoA), as suggested by the slight decrease in its biochemical precursors in treated cells (Supplemental Figure 2). CoA is a pivotal cofactor essential for anabolic pathways involved in lipid, amino acid and carbohydrate metabolism: its augmented levels suggest increased metabolic rate in TAp73 expressing cells.

**Figure 2:**
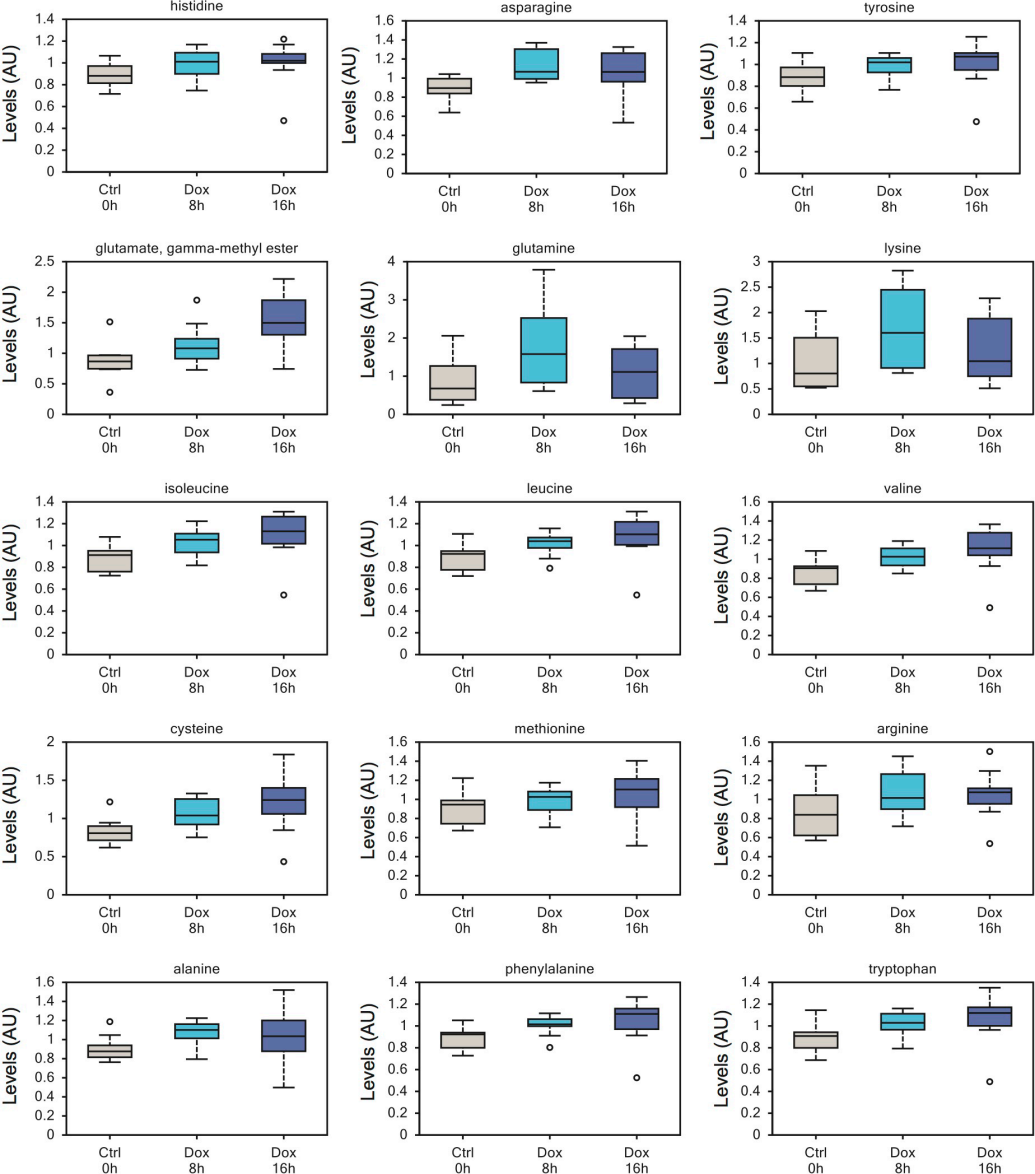
Increased amino acid uptake in TAp73 expressing cells Increased intracellular contents of numerous amino acids after short-time induction of TAp73β, probably reflecting increased uptake from culture medium. This upward trend on intracellular amino acid content reflect an overall increase in anabolism upon induction of TAp73. All metabolomics data are represented as box plot, with upper and lower quartile, max and min distribution values and median value.

Another relevant observation, and one of the most consistent changes, was a significant higher level of amino acids and amino acid metabolites in Dox-treated samples (Figure 2). This can be interpreted as increased uptake of metabolites from the culture media and suggests increased metabolic activity as a result of p73 induction.

**Figure 3:**
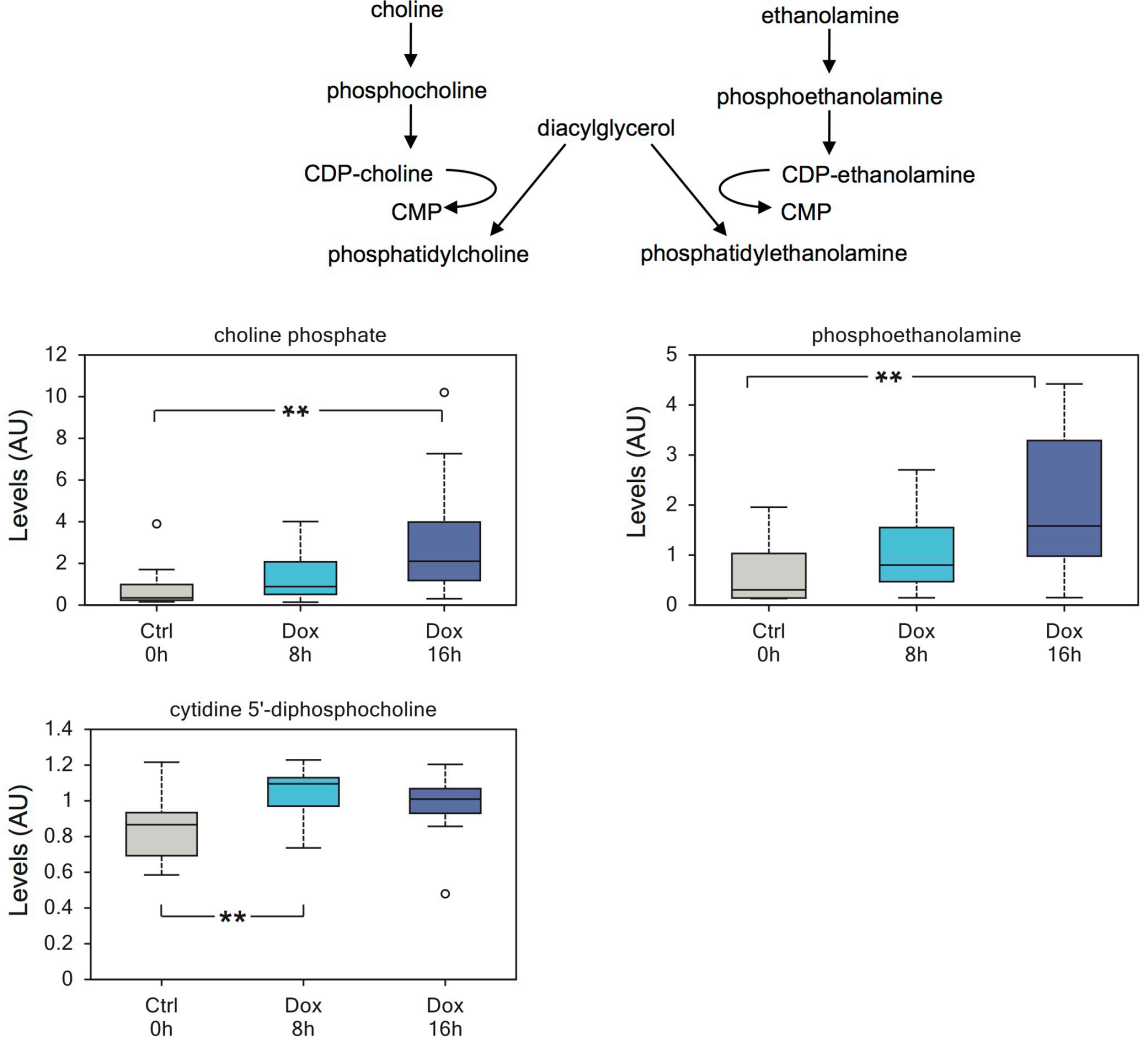
TAp73 regulates biosynthesis of phospholipids Expression of TAp73β induces biosynthesis of membrane lipids, including a substantial increase in phosphoethanolamine. The data also suggest that increased cytidine 5′-monophosphate may partially results from enhanced lipid membrane biosynthesis, through conversion of diacylglycerol to phosphatidylcholine. **p < 0.05.

Moreover, we detected elevated levels of compounds involved in membrane biosynthesis in induced cells, including choline phosphate and phosphoethanolamine (Figure 3).

**Figure 4:**
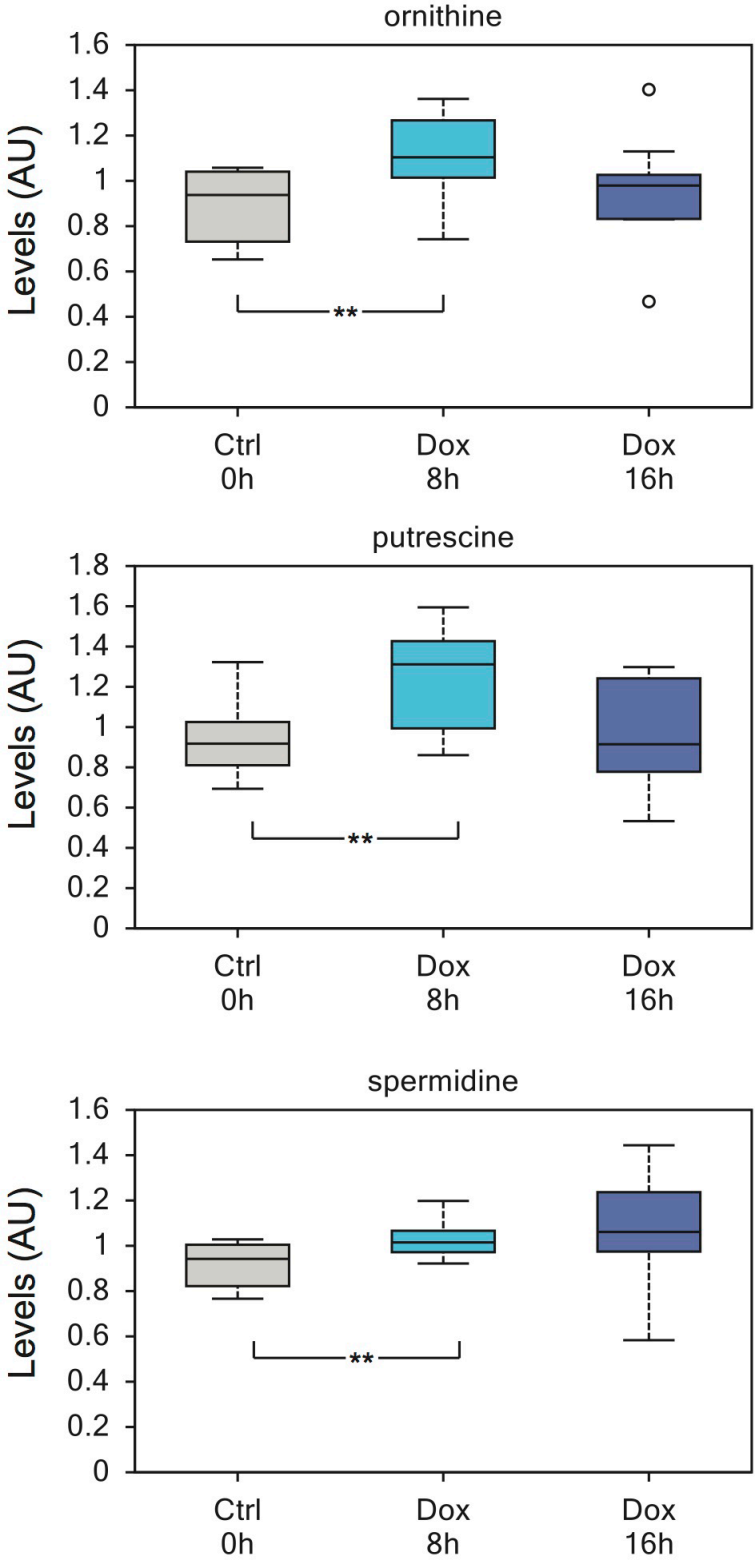
Enhanced polyamines biosynthesis induced by TAp73 Polyamines show a significant increase after 8h of TAp73β induction. Ornithine, putrescine and spermidine are all upregulated. Polyamine expression has been linked to proliferation and polyamine biosynthesis pathway is highly regarded for anticancer therapy. This increase is not maintained at 16h induction and the reason for this is currently unknown. **p < 0.05.

**Figure 5:**
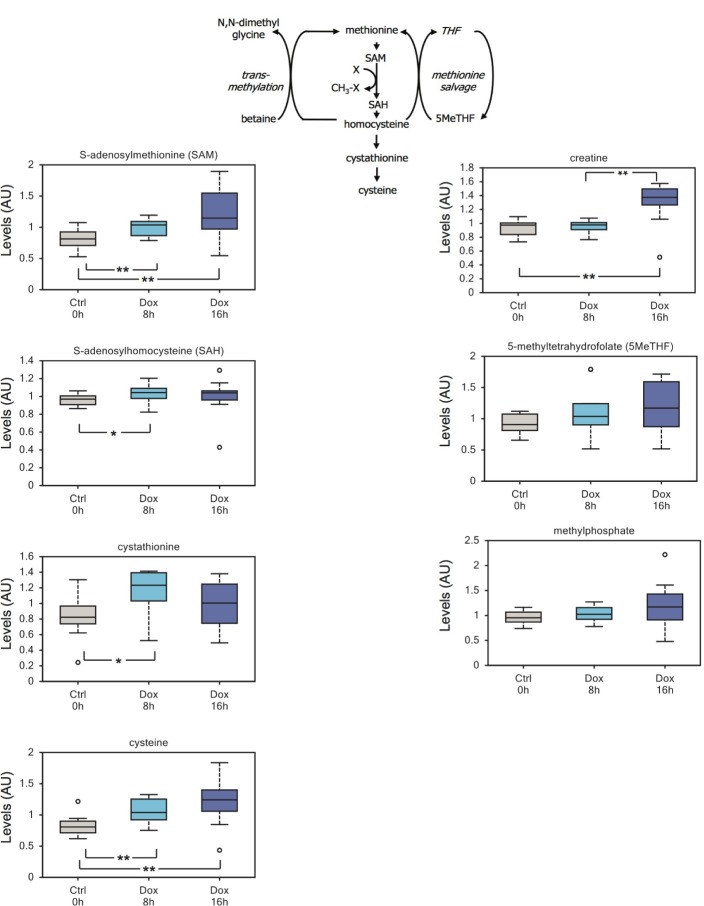
TAp73 expression affects the methionine cycle A significant increment in the methyl-donor SAM correlates with an increase in its demethylated byproducts S-adenosylhomocysteine and cystathionine. Cysteine, the downstream metabolic product of cystathionine, also shows a robust accumulation in induced cells; although we cannot exclude that cysteine accumulation results from increased uptake from growth medium. Finally, we detected an upward trend in the methylated compound 5-methyltetrahydrofolate. Overall these changes suggest that TAp73 might regulate 1-carbon metabolism and epigenetic through availability of the methyl donor SAM. **p < 0.05; *0.05 < *p* < 0.1.

TAp73 also regulates polyamines metabolism, inducing their synthesis. Indeed, ornithine, putrescine and spermidine were significantly upregulated 8h after TAp73 induction (Figure 4) and 5-methylthioadenosine, the byproduct of spermidine and spermine biosynthesis, was also consistently upregulated. Polyamines are essential for cell proliferation and their metabolism is increased by oncogenes [66]. Indeed, targeting polyamines biosynthesis is regarded as a potential anticancer therapy [66]. Thus, these data reinforce the idea that TAp73-mediated metabolic rewiring sustains anabolic pathways.

Arginine metabolism through Urea cycle leads to proline, whose hydroxylation is an important step in collagen synthesis. We observed a significant increase in hydroxyproline and proline-hydroxyproline in induced cells, suggesting extracellular matrix remodeling triggered by TAp73 (Supplemental Figure 3).

Of note, our data also suggest that TAp73 might be involved in epigenetic changes. On one hand, we have described increased levels of acetyl-CoA (Figure 2 and Supplemental Figure 2), by far the main acetyl donor in the cell. On the other hand, we also detected a significant increment in the methyl-donor SAM, which correlated with a more modest increase in its demethylated byproducts S-adenosylhomocysteine (SAH) and cystathionine (Figure 5). Cystathionine is further metabolized to the amino acid cysteine, which shows a robust accumulation in induced cells (Figure 5). Although we cannot exclude that such increase could result from enhanced cellular uptake. Finally, an upward trend in methylated compounds such as 5-methyltetrahydrofolate (5MeTHF) and methylphosphate, supports an overall surge in methylation triggered by TAp73 (Figure 5).

TAp73-expressing cells exhibit a significant accumulation of carnitine conjugates of fatty acids (Supplemental Figure 4). Fatty acids are imported in the mitochondria as acyl conjugates and formation of acyl-carnitines is a limiting step in fatty acids oxidation. The reason for this build-up is unclear. Possibly, TAp73 induction is blocking β-oxidation, as suggested by the observed increment in carnitine β-oxidation intermediates. Alternatively, carnitine conjugates of fatty acids may be used for different metabolic processes (for example membrane biosynthesis).

**Figure 6:**
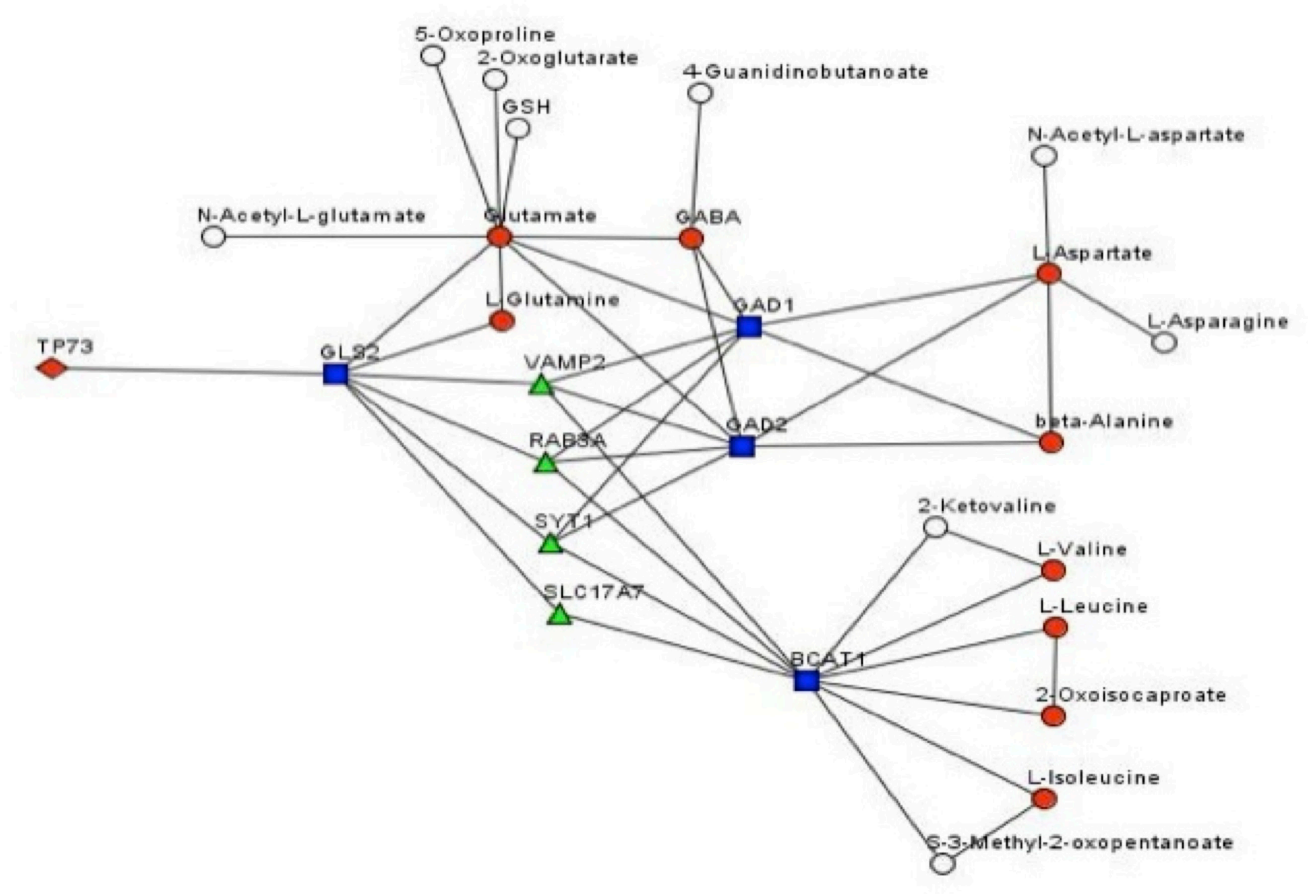
Genes/metabolites network affected by TAp73 Prediction of the gene/metabolite network affected by TAp73 In this network, a node is a gene or a metabolite. Edge between genes means that there is a signalling pathway where the genes are connected (transfer signal, interacts or a part of the same protein complex). Edge between a gene and a metabolite means that the gene product is enzyme known to catalyse the metabolite biotransformations.

To predict the possible gene/metabolite network, underlining the metabolomics effects of TAp73, we implemented a statistical procedure based on our current knowledge of signalling and metabolic pathways. We used all the metabolites resulted upregulated in our analysis, plus KEGG and Reactome databases to construct integral gene-metabolite network [67–69]. We used the integral network to compute the distance between a metabolite and p73. The distance was computed as a minimal number of steps on the network needed to get from p73 to the metabolite. Only 2 measured metabolites (glutamine and glutamate) were located within 2 steps from p73 and both are unregulated (Table 2, and Figure 6). Nine up-regulated metabolites (out of 33) resulted located within 4 steps, while only 7 (out of 160) of not up-regulated metabolites are so closed to p73 (Table 2). Interestingly this computational analysis highlighted the importance in the overall p73-dependent metabolic changes of glutaminolysis. TAp73 regulation on GLS-2 [70, 71], indeed, might widely modulate the entire aminoacids metabolism, beside the simple conversion of glutamine in glutamate (Figure 6). As consequences of GLS-2 regulation, TAp73 appears to indirectly affect aspartate, alanine, valine, leucine and isoleucine, up to 4–5 nodes of distance (Figure 6). However, this prediction is based on the current limited knowledge of p73-metabolic enzyme connections (Figure 6). In conclusion, this analysis confirms p73 effects on cell metabolism and provides a potential explanation at least for part of these effects.

**Table 2:** Summary of metabolic network identified in SaOs-2 cells after TAp73 over expression

Distance	Odds Ratio	Up regulated (33 is a number of upregulated)	All other (160 is a number of all measured metabolites excluding 33 up regulated)	*p*-value (fisher test)
2	N/A (∞)	2 (out of 33)	0 (out of 160)	~0.03
4	6.2	9 (out of 33)	7 (out of 160)	~ 0.001
5	4.5	13 (out of 33)	14 (out of 160)	~0.001
7	3.4	17 (out of 33)	24 (out of 160)	~ 0.01
10	1.2	33 (out of 33)	126 (out of 160)	0.23

The table provides statistics on the number of metabolites that located on integral network within a fixed distance from p73. Odds ratio indicates enrichment of metabolites located within 4 steps to p73 among unregulated metabolites quantitatively: (9/33) / (7/160) ~ 6.2. Fisher test indicates that a probability to get similar or better enrichment randomly selecting 33 metabolites out of 193 measured is less than 1 in 1000.

## DISCUSSION

Oncogenes reprogram metabolism to sustain cell growth, whereas tumor suppressors halt malignancy also by mean of metabolic regulation. The awareness of the intense metabolic rewiring in cancer has stimulated research to unravel its origin and regulation. Recent work has also demonstrated that the p53 family plays an important role in regulating metabolism in different settings [59, 61, 63, 70, 72–76] and suggests that metabolic regulation contributes to the vast complexity of the p53 family functions, including cell death [77–81], redox homeostasis [82], development [83–87], senescence [88–90], aging [91–94] and fertility [95–97]. For this reason, we sought to investigate whether TAp73 regulates metabolism and how this could relate to physiological roles. With this in mind, we performed a high throughput metabolic study of osteosarcoma cells expressing tetracycline-inducible TAp73β. Through careful cell cycle and survival analysis, we were able to establish that induction of TAp73 for 8h and 16h resulted in abundant protein expression and engagement of transcriptional activity without any significant effect on cell proliferation and apoptosis. Hence, we exclude that our results are biased by p73 pro-apoptotic and anti-proliferative activities evident at later time points after TAp73 induction.

Interestingly, we found that expression of TAp73 modulates numerous metabolites with the net result of increasing cellular biosynthetic pathways and promoting glucose consumption and Warburg effect, a metabolic hallmark of cellular transformation [44, 98]. This last effect appears to be in striking contrast to p53-mediated inhibition of glycolytic flux [99]. Moreover, TAp73-expressing cells show increased amino acid uptake and increased levels of acetyl-CoA indicative of increased metabolic rate. Similarly, we detected increased biosynthesis of polyamines, as observed in proliferating and cancerous cells.

Therefore, it appears that, despite TAp73 is able to trigger a molecular program to arrest cell proliferation and induce apoptosis (i.e. upregulation of PUMA and p21) [28], it does promote a metabolic rearrangement that is reminiscent of an opposite, pro-growth and pro-proliferative outcome. This possibility was also suggested by a recently published manuscript reporting that TAp73 sustains proliferation through regulation of the PPP [63]. Du and colleagues show that TAp73-mediated expression of G6PD diverts glucose metabolism towards PPP and production of NADPH, which is required for ROS detoxification [100–104] and ribose for nucleotide biosynthesis and proliferation. Despite we report an increase rate of glucose metabolism, we did not observe evident differences in PPP. We did indeed record a striking increase in nucleotides levels in p73 expressing Saos-2 (manuscript in press), somehow in line with the pro-proliferative activity of TAp73 reported by Du and colleagues [63]. However, different interpretations might be applied to TAp73-depedent nucleotide biosynthesis. Being relevantly involved in DNA-damage response [105–113], TAp73 might also promote nucleotide biosynthesis in order to provide sufficient substrates to the DNA-repair machinery. In their study, Du and colleagues observed a substantial reduction of tumour proliferation in mouse xenograft tumour models after TAp73 knockdown, however this observation was not confirmed in other xenograft models [114], and contradicted by the tumourigenesis of TAp73^−/−^ mice [6].

Overall, these findings bear several implications. Firstly, the operational definition of tumor suppressor might be misleading and might be unable to fully categorize genes. TAp73 regulation of metabolism should be interpreted on the light of its multifaceted activities, which include roles in fertility, development, neurodegeneration and aging [4, 22, 59, 64, 65, 115], beyond its function in cancer. Intriguingly, we and others have shown that lack of TAp73 compromise growth and maintenance of neural stem cells [64, 116]. The findings reported here prompt the intriguing possibility that impaired metabolism might underline the defect in stem cells maintenance after depletion of TAp73 and contribute to the neurodegerative outcome of p73 loss [117–119]. Moreover, we have provided evidence that loss of TAp73 leads to senescence and aging in knockout animals. We were able to correlate these phenotypes with decreased mitochondrial activity, enhanced ROS production and a marked sensitivity to oxidative stress. Nonetheless, the anabolic metabolism promoted by TAp73 might ultimately contribute to prevent accelerated senescence and aging, a possibility that warrants further investigation. This interpretation of the data would also reconcile TAp73 with its repeatedly proved role as tumor suppressor, a function that we have not failed to confirm in the present study, showing induction of p21 and apoptosis upon expression of TAp73.

In this analysis we have identified additional metabolic changes. We report a sustained arginine metabolism in TAp73 expressing cells, which suggests a possible rearrangement of the extracellular matrix (ECM) environment. The recent discovery that p63 suppress cell motility and invasion [120–123], also suggests (i) that p73 might regulates cell motility through ECM rearrangement and (ii) that p63 might share a similar ability to regulate arginine metabolism and, possibly, ECM.

Also worth investigation is the finding of augmented SAM levels, which might link TAp73 to epigenetic rearrangement. If demonstrated, this possibility would reinforce the growing awareness that regulation of metabolism impacts cellular physiological processes, such as epigenetic and gene expression.

Further studies are necessary to address which genetic program underlines TAp73-mediated regulation of cellular metabolism and to understand how this applies to cancer and affects the phenotypes triggered by p73 depletion. Nonetheless, our work has unveiled an unexpected role for TAp73 in promoting anabolism and has clearly established a divergence between p53 and p73 in the regulation of cellular metabolism.

## MATERIALS AND METHODS

### Cells culture

SaOs-2 Tet-On inducible for TAp73 were cultured at 37 °C in 5% CO_2_ in RPMI 1640 medium (Gibco), supplemented with 10% FCS, 250 mM L-glutamine, penicillin/streptomycin (1 U/ml), and 1 mM pyruvate (all from Life Technologies). TAp73 expression was induced by addition of doxycycline 2μg/ml for the indicated time.

### Western blots

Proteins were extracted from cell pellets using RIPA buffer (25mM Tris/HCl pH 7.6, 150mM NaCl, 1% NP-40, 1% sodium deoxycholate, 0.1% SDS) supplemented with phosphatase and protease inhibitor cocktails (ROCHE). Quantification of protein extracts was performed using BCA protein assay from PIERCE. 40μg of protein were boiled for 6 minutes at 90°C and then separated using SDS-PAGE, transferred to nitrocellulose membranes using standard transfer techniques and blocked with 5% milk for 2h at room temperature. Primary antibodies were incubated O/N at 4°C in blocking with gentle agitation. We used rabbit HA (Santa Cruz, Y11), rabbit β-tubulin (Santa Cruz, H-135), rabbit p21 (H-164). Horseradish peroxidase (HRP)-conjugated secondary antibodies (BioRad) and ECL chemoluminescence substrate (PIERCE) were used for final detection.

### Metabolic analysis

TAp73 SaOs-2 Tet-On cell lines were cultured in growing medium and treated for 8h and 16h with doxycycline 2μg/ml to induce TAp73β expression. Control cells were treated with vehicle (PBS) for 16h. Thirty million cells were spun down and pellets were washed once with cold PBS before being frozen in dry ice. All the samples were extracted using standard metabolic solvent extraction methods and analyzed through GC/MS and LS/MS as previously described [124].

### Cell cycle and survival

For cell cycle analysis 500,000 cells were treated for the indicated time with doxycycline 2μg/ml, collected and fixed with ice cold 70% ethanol. After O/N fixing at −20°C, cells were washed in PBS, resuspended in 50μl of 10μg/ml RNase solution (SIGMA) and incubated for 10 minutes at 37°C. 500μl of staining solution (50μg/ml propidium iodide in PBS) was added to the cells, followed by additional incubation 30 minutes at 37°C. Stained cells were analyzed by flow cytometry and at least 10,000 cells per sample were collected. Data were analyzed using CELLQuest acquisition/analysis software.

## SUPPLEMENTARY FIGURES


