# Supplementary material for: TAp73 promotes anabolism
Source: Oncotarget. 2014 Nov 13;5(24):12820–34. doi: 10.18632/oncotarget.2667 (PMC4350352; doi:10.18632/oncotarget.2667)
Supplement: Supplementary file 1 [file oncotarget-05-12820-s001.pdf]

SUPPLEMENTARY FIGURES

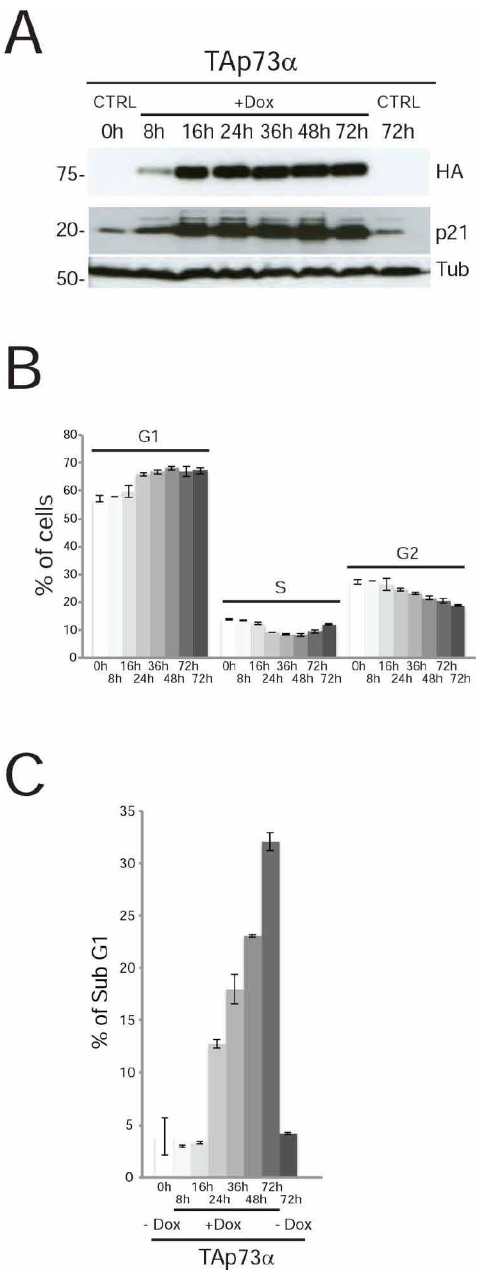

Supplementary Figure S1. (A) TAp73α and p21 expression was assessed by western blot after treatment with doxycycline (2µg/ml) for the indicated time. TAp73 expression was detected using HA antibody to the N-terminal HA tag. Tubulin was used as loading control. Controls were left untreated (0h) or treated with vehicle for 72h to account for changes induced by confluence. **(B)** Cell cycle profile of SaOs-2 cells upon induction of TAp73α determined by PI staining and cytofluorimetric analysis. Controls were like in (**A**). It is evident how induction of TAp73 for 8h and 16h does not affect cell cycle profile of treated cells. Graphs report average of triplicates with standard deviation. **(C)** Cell death assessed by sub-G1 population after PI staining. Induction of cell death is evident only after 24h of TAp73 induction. Data indicate average of triplicates and standard deviation.

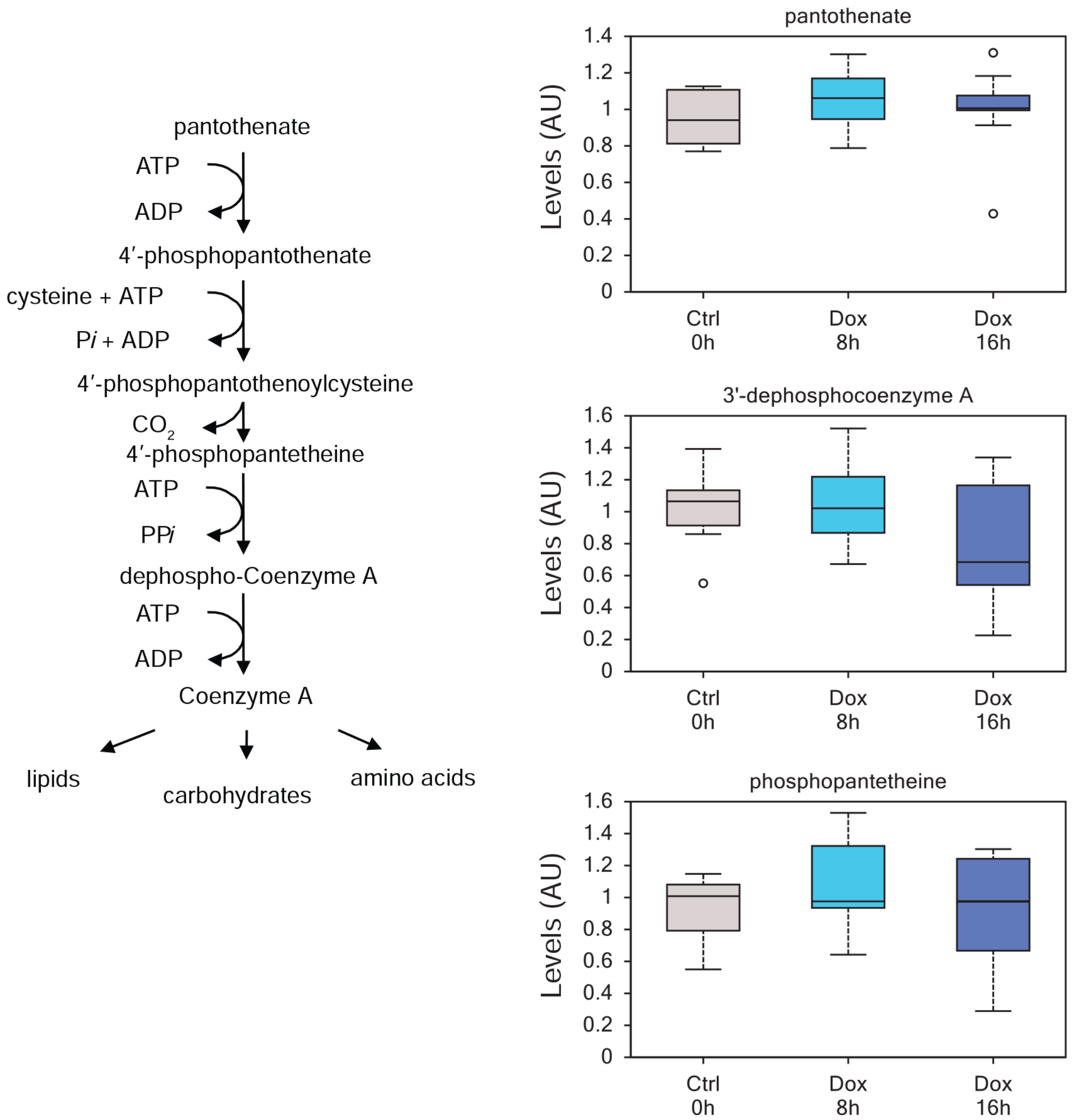

Supplementary Figure S2. The increase in acetyl-CoA levels in induced cells can be the outcome of increased glycolytic rate as reported in Figure 2. Nonetheless, increased acetyl-CoA biosynthesis is suggested by a trend towards reduced levels of acetyl-CoA biosynthetic precursors such as 3’-dephosphocoenzyme A and phosphopanteteine. The overall contribution of newly synthetized acetyl-CoA to the whole cellular pool remains to be established.

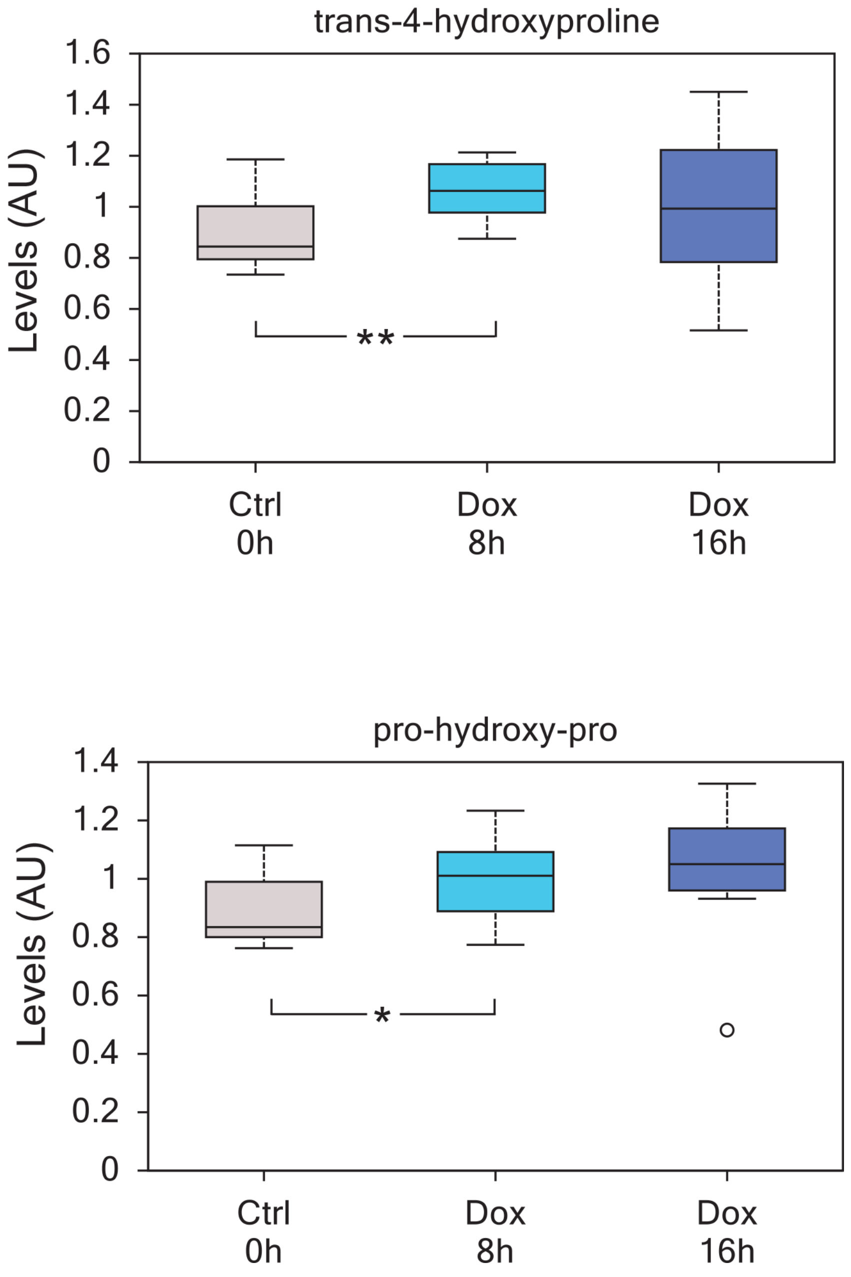

Supplementary Figure S3. Increased hydroxyproline and proline-hydroxyproline levels in induced cells, suggesting extracellular matrix remodeling triggered by TAp73. **$p < 0.05$; *$0.05 < p < 0.1$.

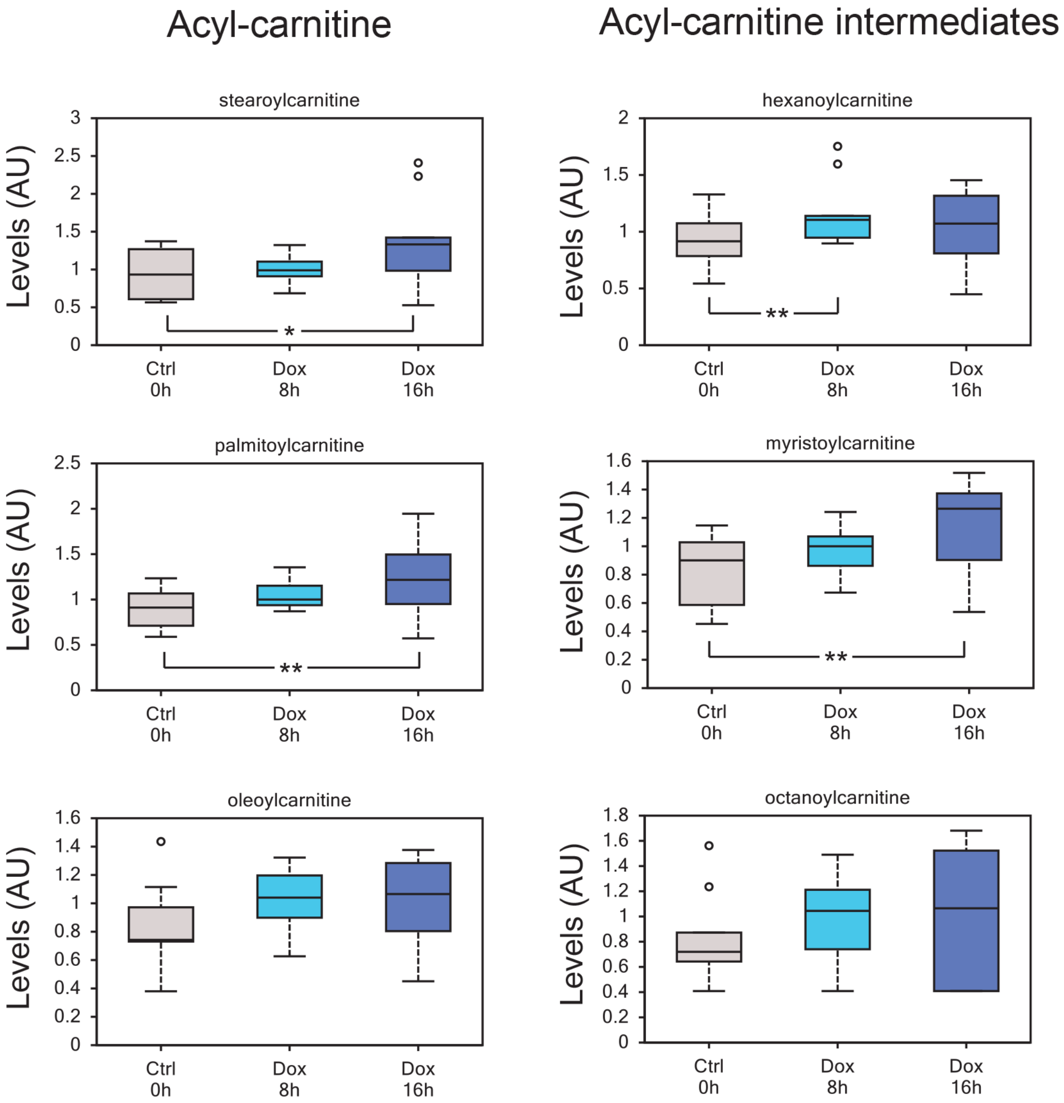

Supplementary Figure S4. An increase in acylcarnitines was detected in TAp73 expressing cells, with palmitoylcarnitine, myristoylcarnitine and hexanoylcarnitine reaching statistical significance. Carnitines are the limiting step in fatty acid oxidation and are responsible for fatty acid import within the mitochondria. Therefore their increase in TAp73 expressing cells could be interpreted either as a reduce import within the mitochondria or as the result of fatty acid mobilization for oxidation. $^{**}p < 0.05$; $^{*}0.05 < p < 0.1$.
